# ‘As long as I have a restroom somewhere […], I am fine’: a qualitative study on the perspectives of peri- and postmenopausal women on the impact of the urinary component of the genitourinary syndrome of menopause (GSM)

**DOI:** 10.1186/s12905-021-01523-x

**Published:** 2021-11-08

**Authors:** Michaela Gabes, Gesina Kann, Julia von Sommoggy, Petra Stute, Christian J. Apfelbacher

**Affiliations:** 1 Institute of Social Medicine and Health Systems Research, Otto-Von-Guericke-University Magdeburg, Dr.-Gessler-Str. 17, 93051 Regensburg, Magdeburg, Germany; 2 Medical Sociology, Department of Epidemiology and Preventive Medicine, University of Regensburg, Regensburg, Germany; 3 Department of Obstetrics and Gynaecology, Inselspital Bern, Bern, Switzerland

**Keywords:** Genitourinary syndrome of menopause, Urinary component, Impact, Qualitative study, Focus group, Menopause

## Abstract

**Background:**

Our aim was to gain insight into the experiences of women suffering from the urinary component of the Genitourinary Syndrome of Menopause (GSM) and to understand the impact of urinary complaints as part of GSM on the lives of affected women.

**Design:**

Qualitative study.

**Setting:**

Online, primary care.

**Participants and methods:**

Postmenopausal women aged from 46 to 85 years reporting vaginal and urinary complaints were recruited to participate in either online or face-to-face focus groups to share their experiences with urinary complaints as part of GSM. Transcripts of sessions were analysed using qualitative content analysis.

**Results:**

One online focus group, one face-to-face focus group and one online-interview were conducted, involving 11 women. Five a priori assumed main themes related to the impact of urogenital symptoms were identified: daily life, emotional well-being, sexual functioning, self-concept and body image, and interpersonal relations and communication. Additionally, two further themes associated with GMS as a clinical condition were inductively found: unmet healthcare needs, including expectations of affected women regarding menopausal symptoms and a lack of adequate health education, and aspects on the personal dealing with the complaints, including personal coping strategies and medical treatment.

**Conclusions:**

This study showed that urinary complaints as part of GSM have, similar to vaginal complaints, negative impacts on the daily life, the emotional well-being, the sexual functioning, the self-concept and body impact as well as interpersonal relations and communication of affected women. We further identified several unmet healthcare needs that should trigger improvements in healthcare.

**Supplementary Information:**

The online version contains supplementary material available at 10.1186/s12905-021-01523-x.

## Background

The introduction of the new term ‘Genitourinary Syndrome of Menopause (GSM)’ in 2014 led to the fact that research is increasingly taking into account the newly added urinary component of GSM [1]. GSM provides a broader perspective on known clinical conditions such as vulvovaginal atrophy (VVA). In addition to the genital and sexual complaints of VVA, GSM focuses also on the urinary component with symptoms such as dysuria, urinary urgency and frequency, incontinence, and/or recurrent urinary tract infections [2].

The negative impact of VVA on the quality of life and sexual health of affected women has already been shown in different studies [3, 4]. In a qualitative study of Huang et al*.* [5] vaginal symptoms in postmenopausal women, such as dryness, soreness, itching, and pain with sexual intercourse, affected everyday activities, the emotional well-being, the sexual functioning, the self-concept and body image, as well as interpersonal relations and communication. We further know that menopause in general is closely related to emotional well-being. In particular, there is a positive association with the occurrence of depressive symptoms during menopause [6, 7]. Furthermore, it has been shown that sexual functioning (e.g. due to less vaginal elasticity) and therefore inevitably sexual activity decreases with menopause [8, 9]. Thus, the impact of VVA or menopause per se has already been studied. However, the impact of the urinary component of GSM has hardly or even not at all been examined so far, albeit recent studies, such as the Italian AGATA study by Palma et al*.*, showed that 36.1% of women with GSM reported dysuria for instance [10].

Therefore, our aim was to close this gap by exploring the urinary component of GSM and its impact on the lives of women with GSM using a qualitative research approach.

## Methods

In qualitative research, focus groups are an economic data collection method since several participants can be reached at one time. The interaction with other participants can activate personal experiences and opinions and therefore create a unique data source [11].

### Patient and public involvement

We did not formally include “real” patients with a clinical diagnosis. However, we included participants having self-reported symptoms. The recruitment process and the conduct of the study is presented in the next paragraph. The results of this study will be disseminated to the study population via social media.

### Participants and procedure

Participants were recruited online via several Facebook groups for postmenopausal women and via flyers in clinics and study centres to conduct either online or face-to-face focus groups. A short screening survey was created via www.soscisurvey.de and posted in the Facebook groups to filter eligible and interested women.

Only postmenopausal women reporting at least one vaginal symptom (vaginal dryness, burning, itching, and/or pain) and one urinary symptom (urinary frequency and urgency, dysuria, incontinence, and/or recurrent urinary tract infections) to assume a GSM symptomatology were included.

Interested and eligible women received the informed consent document and the participant information via e-mail and either a link to the online platform (www.focusgroupit.com) or an appointment for the face-to-face meeting depending on where the participants were located. *Focusgroupit* is a secure platform that was particularly created for the conduction of asynchronously typed online focus groups. The facilitator can post questions and all participants can comment directly beneath the posts.

The online survey was completed by 85 women of whom 78 (91.8%) reported to be postmenopausal. Forty women (51.3%) reported vaginal symptoms and 45 women (57.7%) reported urinary symptoms. Among the vaginal symptoms, vaginal dryness was the most prevalent symptom reported by 30 women and among the urinary symptoms, it was urinary frequency and urgency that was reported by 24 women. The inclusion criteria, at least one vaginal and one urinary symptom, were fulfilled by 25 women (32.1%). Those eligible women had a mean age of 51.6 (± 3.57) years with a range from 44 to 58 years. Of those, only 11 women were interested in the participation in an online focus group and were invited via e-mail. Only 6 women (ID01 – ID06) aged from 46 to 52 years accepted this invitation and discussed the questions.

For the recruitment of face-to-face focus groups, a cooperating gynaecologist (PS) supported the recruitment. Flyers and her e-mail distribution list were used to reach eligible women. Six women responded to the request. Thus, one face-to-face focus group could be established at the Department of Obstetrics and Gynaecology at the Inselspital in Bern, Switzerland. However, only four women (ID07 – ID10) were present. This focus group was led by one researcher, a female PhD student (MG) experienced in the conduct of interviews. After signing the informed consent form, the participants filled out a short survey about demographics, current vaginal and urinary complaints and hormone intake. The mean age of this group was 62.0 (± 16.19) years with a range from 47 to 85 years. All women reported mild to moderate vaginal and urinary symptoms. The focus group discussion lasted approximately 70 min. No field notes were taken.

Furthermore, flyers were given to women participating at the screening for a clinical GSM trial by one of our research partners. Eligible women were asked to contact the research team if they were interested in participating. Only one woman (ID11, age: 53 years) showed interest and was invited to discuss the questions online with the facilitator (MG).

**Fig. 1:**
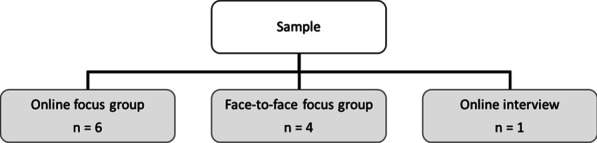
Overview of the included sample

An overview of the included sample is presented in Fig. 1.

**Fig. 2:**
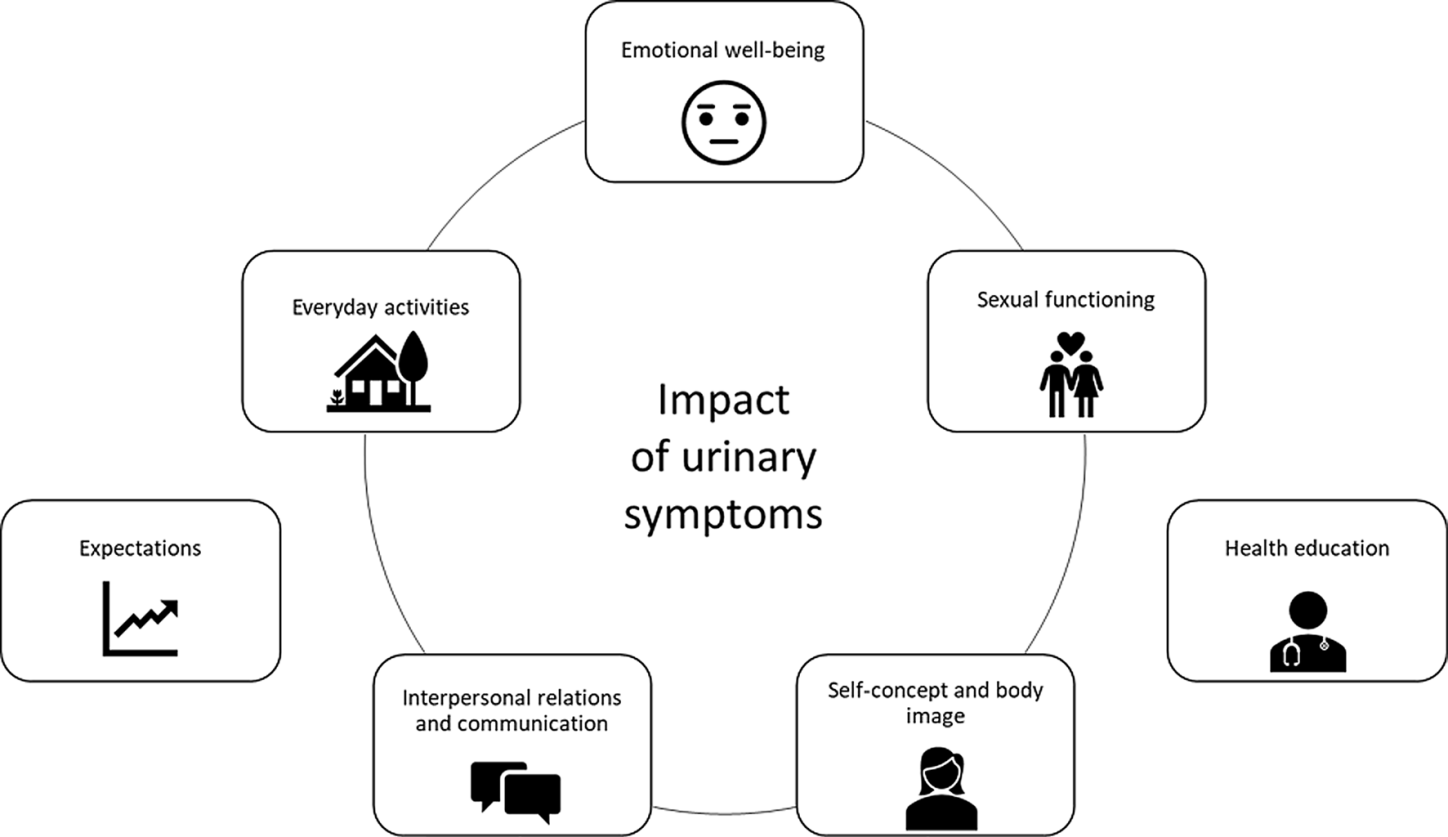
Conceptual model inspired by the results of the focus groups conducted for the development of the Day-to-Day Impact of Vaginal Aging (DIVA) questionnaire

The topic guide for the qualitative data collection was inspired by the conceptual model that formed the basis for the development of the Day-to-Day Impact of Vaginal Aging (DIVA) questionnaire [5]. In that study, it was found that vaginal symptoms have impacts on five domains: everyday activities, emotional well-being, sexual functioning, self-concept and body image and interpersonal relations and communication. Those five domains were included in our topic guide since we expected a negative impact of urinary symptoms on those domains as well. Additionally, we included two further themes, expectations about the development of urinary symptoms and the health education through the treating physician. The conceptual model of our topic guide is presented in Fig. 2. The final topic guide can be found in the Additional file 1 (Appendix A).

The study was approved by the Ethics Committee of the University of Regensburg (file number: 19–1314-101). All methods were performed in accordance with the relevant guidelines and regulations.

### Analysis

Since the online data collection was typed, transcripts were already available. The audiotape of the face-to-face focus group was transcribed verbatim by a professional transcription office (www.abtipper.de). All transcripts were analysed by two independent researchers (MG and GK) using the computer-assisted qualitative data analysis software ATLAS.ti version 8. The researchers conducted the analysis and interpreted the themes. Qualitative content analysis adapted from Schreier [12] was used to analyse the data. Discrepancies in categorization were discussed until consensus was reached. Most of the categories were built deductively from the topic guide. Two further categories inductively arose from the data material. Relevant passages were translated by MG and GK. The research team assumed that data saturation was reached since no new themes were identified in the last online discussion. Additional groups would probably not have modified the thematic framework [13].

## Results

**Fig. 3:**
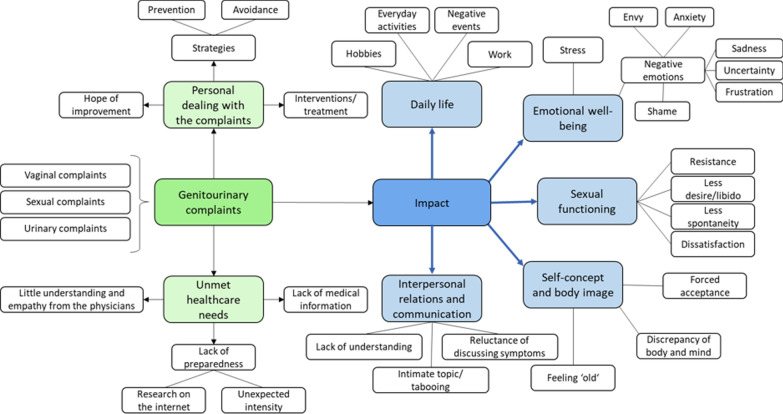
Clustered themes of the qualitative data collection

The five a priori categories about the impact of urogenital complaints were found deductively according to the topic guide. “Everyday activities” became part of the more general category “Daily life”. Two categories arose inductively from the data material: the a priori topics “Expectations” and “Health education” formed the category “unmet healthcare needs” and the category “personal dealing with the disease” was generated inductively from the data (Fig. 3).

### Daily life

All of the participating women stated, that their daily life was affected by their condition. This comprised not only everyday activities such as shopping, but also basic behaviour such as simple hearty laughing. One woman recalled: *“There were and are situations in which I had to force myself to stop laughing. I realized that otherwise I could not hold my urine… and that would have been much more than a drop. That is unimaginable! I love to laugh and I think it's pretty stupid.” (ID06, 49 yrs)* The incontinence made it necessary for the women concerned to prepare for a longer stay away from home (e.g. the grocery shopping in the city). It was described as tedious to constantly keep one’s eyes open for a nearby restroom. The need to use a restroom regularly lead to avoidance of longer car rides and activities such as swimming (e.g. to prevent bladder infections), hiking, travelling or just spending a night at the cinema: *“A week ago I went to the cinema and intentionally I didn't drink much before because they don't take a break in the cinema. And I already felt towards the end […] the pressure, and suddenly it went away […]. I had my whole pants wet.” (ID09, 58 yrs)* Since most women go through menopause before they retire, the complaints also have an impact on work, where it is often even more difficult to deal with them. For those women who had already been retired it was easier to stay at home when their complaints got worse. In order to cope with the symptoms in everyday life most affected women use panty liners or diapers especially for incontinence and tried to drink enough (to avoid infections). Trying to drink enough is always a struggle since one the one hand, it helps to stay hydrated and to avoid infections, but on the other, it is accompanied by an increased urge to urinate. One woman reported to always have “emergency pants” with her.

### Emotional well-being

Being asked which emotions were provoked by the complaints, one woman replied: *“Fears about what is coming towards me. The fears are always there and also worries associated with them.” (ID02, 48 yrs)* Anxiety was only one of six dominant negative emotions identified during the coding process. The other five emotions were shame, envy, uncertainty, frustration and sadness. The latter was often mentioned in connection with sexual functioning. Uncertainty was closely linked with anxiety. Shame was often associated with situations in daily life, mainly caused by incontinence, that were experienced as very embarrassing for affected women: *“The other day I wet the bed! I dreamt that I was going to the bathroom and so I let go. […] That haunted me for days!” (ID05, 50 yrs)* The constant thinking about the complaints often led to mental stress. Affected women described themselves as being nervous and frustrated because of their symptoms: *“I find it very stressful when there is a constant feeling of pressure, regardless of whether I can urinate little or a lot. The pressure makes me highly nervous.” (ID01, 52 yrs)* One woman reported to be envious of those women having no complaints: *“I have to admit that I am a little envious of the ladies who have no or hardly any complaints.” (ID06, 49 yrs).*

But not for all affected women menopause was a “nightmare”. Some women also found a more positive way to deal with their complaints and seemed to be a bit more optimistic. This is discussed in more detail in the section of “Personal dealing with the complaints”.

### Sexual functioning

Another issue that was frequently mentioned, especially by women in a relationship, was the impact of their complaints on sexual functioning. While single women reported that e.g. the loss of their libido was even felt as a relief, it still important for women in a relationship. Having complaints not only influenced the women themselves, but also their partnership which includes a loss of intimacy with their partner and a decreased quality in their sexual relation: *„I think if I were alone, it would stress me less, yes. Because I would think, yes, this is my need and if it’s not there, it’s just not there. But it is not only my need, it is also a need of my husband. It is a need that comes with being together, yes.” (ID10, 47 yrs).*

Due to urogenital symptoms such as incontinence, vaginal dryness or soreness, loss of libido and a permanent fear of subsequent infections, sexual intercourse is no longer experienced as pleasant and satisfying by those affected. It is rather associated with embarrassing experiences and worries: *“For a few months now, my libido has been pretty much zero, which I don't know from me. The feeling during sexual intercourse is completely different. It feels partly sore and everything is very sensitive. […] My bladder has to be emptied first, otherwise more than one drop comes out, which is also very unpleasant, also because of the smell. I have to admit that I even cried once "afterwards" because the feeling is completely different than before. More unpleasant than beautiful. The certainty of orgasm is no longer there. Often nothing at all happens to me. This is really frustrating for me. And the fear of a final bladder infection always accompanies me. It's really mentally stressful.” (ID06, 49 yrs)* Furthermore, women described their sexual life as more complicated and less spontaneous. This resulted in a need for more romantic time with their partners. It was felt to be necessary to be understood by their partner to maintain a level of intimacy.

Some women noted that they had become more reluctant in their sexual relation with their partner. Other described that even though they did not feel any pain, they didn’t feel any pleasure either and thus, had to overcome themselves to be sexually active: *“I also have to overcome myself sometimes. I also find it important that I don't just say that I have no more desire or something like that. […] In my heart I have the desire to love my husband. But somehow the body no longer comes along. That's the hard part.” (ID10, 47 yrs).*

In order to deal with their complaints, affected women often talked about strategies that they had established to prevent infections (e.g. taking a shower after sex). One measure often mentioned to prevent urine loss during intercourse and vaginal or urinary infections afterwards, was the use of the restroom before and after sexual intercourse.

### Self-concept and body image

Three main aspects of self-concept and body image caused by the complaints were voiced by the women: a feeling of being ‘old’, forced acceptance and a divergence of body and mind.

One woman described the symptoms accompanying menopause as “*signs of aging that are difficult to influence*” *(ID11, 53 yrs).* Women often complained of a urine odour due to incontinence that made them feel uncomfortable and embarrassed and reinforced the feeling of being ‘old’. Affected women are oftentimes surprised by the unexpected changes of their bodies, and felt like being forced into acceptance: *“I also think that when you get older, your body changes and it is also normal that you miss it, that you are not as you were before when you were really young and good and comfortable.” (ID07, 58 yrs)* This was intensified especially in women who are in a relationship. They felt a divergence between their body and mind. Although in their minds they still wanted to give love to their partner and be sexually intimate, their body no longer allowed this to happen in the way it used to. One woman called her own body an “enemy”. Another woman described a loss of inner balance explaining: *“That is what made me the saddest. To sense that my heart wants to, but my body can no longer keep up. […] Because my heart is full of love for my husband, […] but the balance in my body is no longer right. And to find somewhere a way to maintain this balance again, to give and receive love and so on. That is a challenge for me.” (ID10, 47 yrs)* These aspects generate a feeling of great discomfort in and with their own bodies in affected women.

### Interpersonal relations and communication

Affected women often had inhibitions to discuss their problem because of its intimate nature: *“It is so intimate and private. […] So, it's not only the vagina that hurts, it also hurts in the heart. And I don't find it so easy to talk about that.” (ID10, 47 yrs).*

Even though the first step always means overcoming oneself and opening up, this step was mentioned to be less difficult in a trustful partnership, the family environment or with close friends: *“I talk about it with my partner. The complaints are not to be overlooked. He is very sympathetic, which is really good. I also discuss the topic with my close friends. They suffer from bladder infections as well and can understand me very well.” (ID06, 49 yrs)* The understanding of others was often experienced as a relief.

Half of the participating women were confronted with a lack of understanding or even tabooing when trying to talk about their condition, not only with their friends but also in the society as a whole: *“I live alone and have decided to keep my complaints to myself in the future. No matter with whom, neighbourhood, relatives, acquaintances, doctors …everywhere no understanding!” (ID01, 52 yrs).*

Three women mentioned the use of the internet and especially internet forums for menopausal women to exchange experiences. One of them concluded that the anonymity provided by these forums, made it easier for those affected to deal with their complaints, to talk about them and to get help: *“I didn't know anything about menopause, I came across it in an internet search. I was looking for help there because of sudden bad, unexplainable complaints.” (ID01, 52 yrs).*

### Unmet healthcare needs

Most women reported that they had neither expected urogenital symptoms with such an intensity, nor at such an early stage, given the fact that some of the participating women were only in the forties: *“So, I was completely overwhelmed by the menopause. Of course, I knew about it, but I didn't know that it could affect me that way. I wasn’t prepared for the extent of the symptoms.” (ID02, 48 yrs)* Another woman said: *“I didn't expect these complaints so soon. I knew that urinary incontinence is likely in old age. I did not think that I could get these complaints already in the mid/late forties.” (ID06, 49 yrs)* On the one hand, affected women saw a general lack of discussion about possible symptoms as a potential reason for their own ignorance. On the other hand, this problem seemed to be aggravated by a lack of understanding and empathy by their attending physicians: *“I was also badly informed, I had no idea. I had very, very heavy bleedings and my doctor just gave me some pills. But [he did not mention] […] that this could have a connection to the fact that menopause is imminent.” (ID09, 58 yrs)* Furthermore, affected women usually had to address their complaints on their own initiative, which is often an overcoming, as described above. When they had finally decided to seek help, some affected women claimed that their concerns had hardly been discussed, they had been hastily referred to a urologist, or they had even been confronted with condescending comments by the doctor: *“When I talked to the doctor, it took me a lot of courage, because I think this is something intimate, something personal. […] And she [the gynaecologist] shrugged it off and said maybe you are a bit stressed and so on. Yes, of course it is stressful when it [the libido] suddenly disappears from one day to the next.” (ID10, 47 yrs)* When asked what they would expect from their physicians or what constitutes good medical advice for them, most women had a clear idea: *“Now that I am in menopause, I would have liked to have an informative conversation with my gynaecologist about the menopause. I know that "only" one third of women has severe symptoms, one third has mild symptoms and one third has none at all. Nevertheless, I think it is important that women in their late thirties are given a brief explanation of what they might have to expect.” (ID06, 49 yrs)* But not only information about possible physical complaints is needed: *“I think that a competent consultation that is looking at the whole individual and sometimes also addresses the psychological perspective like ‘How are you doing?’ is extremely important.” (ID10, 49 yrs).*

### Personal dealing with the complaints

Another topic that arose during the discussions was the personal dealing or coping with the complaints. Some of these individual coping strategies have already been mentioned in the previous sections “Daily life” and “Sexual functioning”. The strategies reported in this study can be divided into two groups: prevention strategies, such as staying hydrated, using panty liner or urinating before and after sexual intercourse, or avoidance strategies, such as avoiding longer car rides or spontaneous sexual activity. Both, prevention and avoidance strategies were perceived as restricting quality of life.

Two other aspects in dealing with urogenital complaints emerged during the discussion. The first one was medical treatment, especially hormone therapy. Finding the right combination and doses of medication can be stressful for some affected women: *“In the beginning it was difficult to adjust to it [the medication], until it somehow worked a little bit. But now I really feel much more comfortable.” (ID10, 47 yrs)* The second one was the inner attitude. Even though a lot of negative emotions connected with the complaints were mentioned, some women tried to stay positive and make the best of it: *“I am not giving up hope that one day I will feel almost the same again as before menopause.” (ID06, 49 yrs)* Another woman added: *“I also think your own attitude is important. So, for me now, for example, it is gratitude [to have medication].” (ID08, 85 yrs).*

These aspects can give affected women a sense of control over their body and life, at least to a certain degree or as one woman concluded: *“As long as I have a restroom somewhere and my water, I am fine.” (ID09, 58 yrs).*

## Discussion

This qualitative study showed that urinary complaints as part of GSM have negative impacts on the daily life, emotional well-being, sexual functioning, self-concept and body image, and interpersonal relations and communication of affected women. With our quantitative screening survey, we could confirm that vaginal dryness was the most prevalent symptom of postmenopausal women [14]. Among the urinary symptoms, urinary frequency and urgency were the most reported symptoms. In the qualitative data, many women reported a lack of preparedness. They did not expect to develop complaints of this intensity and not that early. Furthermore, a lot of unmet healthcare needs were mentioned during the data collection. Especially a lack of medical information and little understanding and empathy from the physicians were deplored. This finding was supported by a recent Australian in-depth qualitative study that found a lack of knowledge of the long-term consequences of menopausal symptoms [15]. It was up to the women to address their complaints to their attending doctors. In dealing with their complaints, a range of prevention or avoidance strategies was reported by the women. Some made use of medical treatment such as hormone therapy and others simply hoped for improvement. All of these findings are consistent with those of Huang et al*.* [5] indicating that not much has changed in dealing with urogenital complaints within the last decade.

In this study, we had both an online data collection and a face-to-face focus group session. On the one hand, the online data collection reduced geographical limitations, time constraints, and travel and transcription costs. Due to its anonymous character, it provided an ideal platform for highly sensitive, shameful and tabooed topics. However, on the other hand, virtual data collection demands certain skills from the study population. It may exclude those who lack internet access or those who have lack online literacy. We decided to conduct the online focus groups asynchronously. This allowed for a wider participation since participants were not committed to any particular time. However, asynchronous focus groups may not be sufficiently responsive since participants are not interacting immediately. The conversational dynamics unique for a focus group may suffer [16]. Furthermore, there is no voice intonation or any other non-verbal language while typing texts. However, typing gives more time to think about the questions and to answer more comprehensively.

### Strengths and limitations

Our sample size was limited. It was very hard to recruit postmenopausal women having both vaginal and urinary complaints and an interest in participating in the study. Those difficulties support the suspicion that vaginal and urinary complaints during menopause are still a tabooed topic what was also confirmed by the participants. For this reason, we initially started with the recruitment for online focus groups. A theoretical saturation of the data could be only assumed, but not confirmed since the data collection had to be stopped because of recruitment problems. Furthermore, we only included women with self-reported symptoms and not a clinical diagnosis.

### Implications for further research and clinical practice

Since the participating women reported many unmet healthcare needs, these findings can be used as an impetus for improvements in the healthcare system. More information about the aetiology of the symptoms and the different treatment options is needed in order to avoid a feeling of being at mercy of those physical changes [15]. Having an open ear and taking the women’s complaints seriously can already achieve so much. The physicians asking proactively for urogenital complaints is a simple step to reduce shame and inhibitions [17]. Making clear to the women that that they do not have to deal with their complaints alone, can encourage them to report their symptoms and thus contribute to reduce the number of underdiagnosed affected women as it is still the case [2, 18]. According to the holistic model of care for healthy menopause, a women should be always seen as a whole, beyond her hormonal, reproductive and physiological health [19]. In clinical practice, there is often little time to examine each patient sufficiently. In order to gain a quick insight into the patient’s perspective, there are many patient-reported outcome measures, such as the Menopause Rating Scale (MRS) [20] or the Day-to-Day Impact of Vaginal Aging (DIVA) questionnaire [21], that can be completed by the patients while they are waiting and used as a door opener for a trustful conversation.

There is currently no high-quality patient-reported outcome measure available that covers all three components (genital, sexual, urinary) of GSM [22]. This qualitative study shows that there is a need for a tool that is able to capture the impact of the urinary component of GSM. This qualitative data material can be used to generate items for this third part of GSM. The first two components (genital and sexual) are already covered by the DIVA questionnaire that has shown sufficient measurement properties in multiple studies^21,23,24^.

## Conclusion

This study showed that urinary complaints as part of GSM have, similar to vaginal complaints, negative impacts on the daily life, the emotional well-being, the sexual functioning, the self-concept and body impact as well as interpersonal relations and communication of affected women. We further identified several unmet healthcare needs that should trigger improvements in healthcare.

## Supplementary Information

**Additional file 1. Appendix A**. Topic guide.

## Data Availability

The transcripts used and analyzed during the current study are available from the corresponding author on reasonable request.

## Abbreviations

DIVA: Day-to-day impact of vaginal aging questionnaire
GSM: Genitourinary syndrome of menopause
MRS: Menopause rating scale
VVA: Vulvovaginal atrophy
