# Supplementary material for: ‘As long as I have a restroom somewhere […], I am fine’: a qualitative study on the perspectives of peri- and postmenopausal women on the impact of the urinary component of the genitourinary syndrome of menopause (GSM)
Source: BMC Womens Health. 2021 Nov 8;21:391. doi: 10.1186/s12905-021-01523-x (PMC8573922; doi:10.1186/s12905-021-01523-x)
Supplement: Supplementary file 1 — Additional file 1. Appendix A. Topic guide. [file 12905_2021_1523_MOESM1_ESM.pdf]

Appendix A Topic guide

Topics	Questions	Triggers
Personal experiences with urological complaints	Which urological complaints (increased urge to urinate, painful and/or difficult urination and increased urinary tract infections) have you suffered from since the beginning of the menopause and which of these complaints limits you the most?	
Everyday activities	To what extent do your urinary complaints interfere with your everyday activities? Which activities are especially affected?	Going for a walk, sitting for a long period of time, cycling, choice of clothes, rituals to relieve/tolerate the symptoms
Emotional well-being	To what extent do your urinary complaints affect your emotional well-being? How do you feel because of your complaints?	Depression, frustration, shame, fear, worries
Sexual functioning	How much do your urinary problems affect your sexual functioning? Are you still interested in sexual activities? Do you still experience satisfaction during any sexual activity? Are you satisfied with your sex life? What do you wish for?	Feelings of guilt, avoidance strategies, covering up the complaints
Self-concept and body image	How do your urinary complaints influence your body image? How do you perceive yourself?	Feeling ‘old’, decreasing “femininity”, social pressure
Interpersonal relations and communication	To what extent do your urinary complaints affect your interpersonal relations (friendships, partnership, family)? Do you talk about your complaints? If yes, with whom? If not, why not?	Overcoming, fear of being laughed/not being taken seriously, shame, isolation
Expectations regarding urinary complaints	Did you expect to develop such complaints and why? To what extent did you know about these symptoms at the beginning of the menopause? What do you think are the causes of your symptoms?	
Health education by the attending physician	Did you find it difficult to discuss your complaints with a physician? Did you have the feeling that your physician was familiar with your complaints? Did you feel taken seriously?	
